# The Fungal bZIP Transcription Factor AtfB Controls Virulence-Associated Processes in *Aspergillus parasiticus*

**DOI:** 10.3390/toxins9090287

**Published:** 2017-09-16

**Authors:** Josephine Wee, Sung-Yong Hong, Ludmila V. Roze, Devin M. Day, Anindya Chanda, John E. Linz

**Affiliations:** 1 Department of Food Science and Human Nutrition, Michigan State University, East Lansing, MI 48824, USA; lunohong@yahoo.co.kr (S.-Y.H.); devinday108@gmail.com (D.M.D.); 2 Department of Microbiology and Molecular Genetics, Michigan State University, East Lansing, MI 48824, USA; 3 Center for Integrative Toxicology, Michigan State University, East Lansing, MI 48824, USA; 4 Department of Plant Biology, Michigan State University, East Lansing, MI 48824, USA; roze@msu.edu; 5 Department of Environmental Health Sciences, University of South Carolina, Columbia, SC 29208, USA; achanda@mailbox.sc.edu

**Keywords:** aflatoxin, AtfB, virulence-associated processes, oxidative stress, mycotoxin

## Abstract

Fungal basic leucine zipper (bZIP) transcription factors mediate responses to oxidative stress. The ability to regulate stress response pathways in *Aspergillus* spp. was postulated to be an important virulence-associated cellular process, because it helps establish infection in humans, plants, and animals. Previous studies have demonstrated that the fungal transcription factor AtfB encodes a protein that is associated with resistance to oxidative stress in asexual conidiospores, and AtfB binds to the promoters of several stress response genes. Here, we conducted a gene silencing of AtfB in *Aspergillus parasiticus*, a well-characterized fungal pathogen of plants, animals, and humans that produces the secondary metabolite and carcinogen aflatoxin, in order to determine the mechanisms by which AtfB contributes to virulence. We show that AtfB silencing results in a decrease in aflatoxin enzyme levels, the down-regulation of aflatoxin accumulation, and impaired conidiospore development in AtfB-silenced strains. This observation is supported by a decrease of AtfB protein levels, and the down-regulation of many genes in the aflatoxin cluster, as well as genes involved in secondary metabolism and conidiospore development. Global expression analysis (RNA Seq) demonstrated that AtfB functionally links oxidative stress response pathways to a broader and novel subset of target genes involved in cellular defense, as well as in actin and cytoskeleton arrangement/transport. Thus, AtfB regulates the genes involved in development, stress response, and secondary metabolism in *A. parasiticus*. We propose that the bZIP regulatory circuit controlled by AtfB provides a large number of excellent cellular targets to reduce fungal virulence. More importantly, understanding key players that are crucial to initiate the cellular response to oxidative stress will enable better control over its detrimental impacts on humans.

## 1. Introduction

The basic leucine zipper (bZIP) transcription factors are highly conserved in eukaryotes, and play critical roles in stress response pathways. These proteins are able to form homodimers or heterodimers, and bind specific DNA sequences to regulate the expression of genes involved in cellular responses to oxidative stress. For example, the evolutionarily conserved bZIP protein, Nrf2, is known to form heterodimers with ATF4 or MAF, and bind to antioxidant response elements (ARE) found in more than 200 promoters in the mammalian cells *Drosophila*, *Caenorhabditis elegans*, and yeast [1,2]. Signaling pathways that participate in the activation of Nrf2 have been implicated in various pathological conditions such as inflammation, cardiovascular dysfunction, neurodegenerative disorders, premature aging, and cancer [3]. Thus, one of the major objectives of studying the regulatory network that coordinates the cellular response to stress is to enable better control of the detrimental effects of cell dysfunction following oxidative insult.

In filamentous fungi, the modulation of bZIPs including AP-1, AtfA, AtfB, FlbB, JlbA, MeaB, NapA, and RsmA mediates responses to oxidative stress [2,4,5,6,7,8,9,10,11,12]. For example, Apyap-1, a member of the Yap1 family, was associated with the timing and level of conidiospore formation and aflatoxin biosynthesis, as well as susceptibility to oxidative stress in *A. parasiticus* [5]. bZIP proteins that belong to the ATF/CREB family, such as AtfA and AtfB, were first characterized relative to their roles in the stress tolerance of *Aspergillus oryzae* conidiospores [4,6]. Furthermore, an *A. nidulans* putative yAP-like (sub-family of AP-1) bZIP protein, RsmA was demonstrated to be involved in fungal defense against fungivorous insects [7,13]. Although numerous studies established that bZIP proteins help regulate stress response, the extent and the functional significance of the bZIP regulatory network have not been characterized in detail.

In *A. parasiticus*, we previously showed that a 32 kDa protein (p32) binds to a cAMP response element binding site (CRE1) in an early aflatoxin pathway gene, *nor-1*. Mutation in CRE1 (TGACATAA) prevented *nor-1* up-regulation by the cAMP/PKA signaling pathway in response to exogenous cAMP [14]. We also showed that p32 (later described as the 34 kDa AtfB protein) physically interacts with the zinc binuclear cluster transcription factor and positive aflatoxin pathway regulator called AflR [14,15]. Aflatoxin biosynthesis involves approximately 27 genes clustered in a 70-kb region in the *A. parasiticus* genome, and AflR is required for the expression of most aflatoxin genes under aflatoxin-inducing conditions [16,17]. Nucleotide sequence analysis demonstrated that the p32 protein contains a leucine zipper and DNA basic protein domains typical of bZIP proteins, and exhibits 96% identity to the *A. oryzae* bZIP protein AtfB. We further demonstrated that under aflatoxin-inducing conditions, AtfB binds to promoters of seven aflatoxin genes (including *nor-1*) harboring cyclic AMP response elements (CRE), and does not bind to promoters that do not contain CRE sites. Under aflatoxin non-inducing conditions, this binding effect is almost abolished [2]. VeA, a global regulator of secondary metabolism, is required for AtfB to bind promoters of aflatoxin genes. Although evidence supports AtfB binding to promoters in the aflatoxin gene cluster, and VeA likely plays a role in this process, details of how AtfB regulates gene expression remain unclear. The goal of the current study was to characterize the role of AtfB in the regulation of specific target genes at the level of transcriptional activation. The ability to monitor AtfB function and the expression of targets in the AtfB network will provide a predictive model to study the molecular mechanisms that control aflatoxin biosynthesis and secondary metabolism.

In previous work, we proposed that AtfB integrates secondary metabolism and the cellular response to oxidative stress [2]. Although it was clear that this association exists, it was not known whether AtfB directly impacts aflatoxin synthesis and secondary metabolism. The current study demonstrates that AtfB is a master regulator of at least three functional networks, including those involved in secondary metabolism (SM), stress response (SR), and conidiospore development (CD). SM, SR, and CD are important virulence-associated cellular processes in *A. parasiticus*. Furthermore, AtfB regulates novel targets involved in actin cytoskeleton arrangement/transport and cellular defense pathways. These data provide new insights into the functional significance of cellular pathways that contribute to fungal virulence at the molecular level.

## 2. Results

### 2.1. Molecular Analysis of AtfB-Silenced Strains

**Figure 1:**
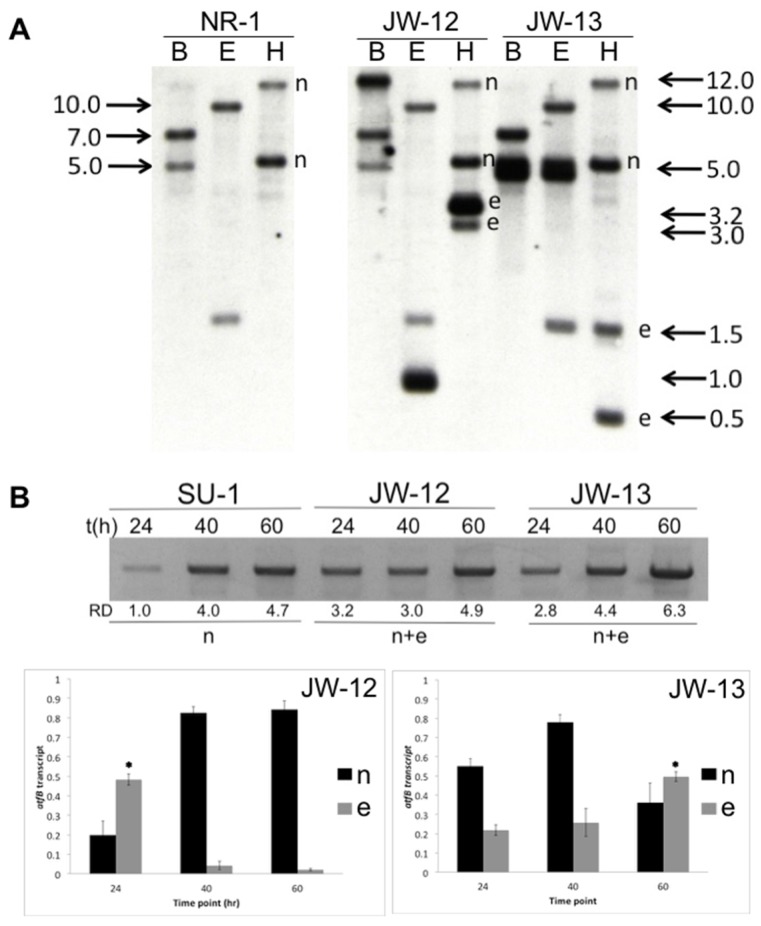
AtfB-silenced strains demonstrate evidence of RNAi-based gene silencing via “quelling”. (**A**) Molecular analysis of AtfB-silenced strains, JW12 and JW13. Strains NR-1, JW12, and JW13 were grown in YES liquid medium for 40 h, and genomic DNA was extracted for Southern hybridization analysis with an *atfB* probe (see Methods; Supplement Figure S1). DNA was digested with *Bam*HI (B), *Eco*RI (E), or *Hind*III (H) and analyzed using standard methods. Southern analysis of NR-1 (recipient and control strain) derived from wild-type SU-1 demonstrates at least two copies of AtfB by high stringency Southern hybridization analysis (designated ‘n’ for native). Additional bands present in the *Hind*III digest (restriction site inserted into the silencing construct) are designated ‘e’ for ectopic integration. (**B**) (top panel) Time course analysis of total *atfB* transcript in SU-1, JW-12, and JW-13. Primers were used to amplify the full length 957 bp *atfB* transcript at 24, 40, and 60 h. R.D., relative density as measured by ImageJ densitometry software analysis (Schneider et al., 2012). n: native transcripts expressed by two wild-type AtfB copies; n + e: native and ectopic (transgene) transcripts produced and expressed. A *Hind*III-cutting assay was conducted on amplified full-length *atfB* transcripts obtained from strains SU-1, JW-12, and JW-13 after 24 h, 40 h, and 60 h of growth (see Methods). We calculated the ratio of native *atfB* transcript (n) to total *atfB* transcript (native AtfB transcript [n] + transgene AtfB transcript [e]), as well as the ratio of the transgene *atfB* transcript (e) to total *atfB* transcript (n + e) expressed in AtfB-silenced strains at 24 h, 40 h, and 60 h. The ratios of wild-type *atfB* transcript (n) to *atfB* transgene transcript (e) in JW-12 (bottom left panel) and JW-13 (bottom right panel) are represented as bar graphs calculated based on densitometry analysis. The asterisks (*) denote the time point where the transgene transcript is expressed at its highest levels.

Ninety-three *niaD* positive transformants were obtained from the transformation of the AtfB knockdown construct (Figure S1) in the control strain, NR-1. PCR was conducted to determine the genotype of these transformants (see Methods, Figure S2), which resulted in two isolates that were analyzed further, designated JW12 and JW13. NR-1 (the recipient and co-transformation control strain), JW12, and JW13 were grown in yeast extract supplemented with sucrose (YES) liquid medium, and genomic DNA was extracted and analyzed by Southern blot under high stringency conditions. Genomic DNA isolated from NR-1 was digested with *Bam*HI, *Eco*RI, or *Hind*III, and the resulting pattern of fragments suggested the presence of at least two copies of *atfB* (Figure 1A). A more intense band was observed at 7.0 kb in the *Bam*HI digest, and a less intense band was observed at 5.0 kb. These two bands observed in the recipient and control strain NR-1 appeared intact at the same location in AtfB-silenced strains (designated ‘n’ in Figure 1A), which indicated that the wild-type *atfB* gene copies (native, ‘n’) were not replaced by the *atfB*-silencing construct during co-transformation. Similar results were observed with the other two restriction enzymes. However, based on restriction fragment patterns with all three restriction endonucleases, we did observe ectopic integration (non-native recombination designated ‘e’ for ectopic in Figure 1A) of the *atfB*-silencing construct at different locations in AtfB-silenced strains. For each restriction enzyme, band intensity of ‘e’ was approximately three times higher than that of ‘n’, which suggested a multiple tandem integration of the *atfB* transgene at that non-native locus. Furthermore, the presence of a new *Hind*III-site inserted into the AtfB-silencing construct was observed in the *Hind*III digest of genomic DNA of AtfB-silenced strains. So, the multiple tandem integration of the AtfB-silencing construct integrated at different ectopic sites in the AtfB-silenced strains.

To test the hypothesis of post-transcriptional gene silencing (PTGS), we amplified a full-length *atfB* transcript by semi-quantitative PCR to make AtfB-specific cDNA using total RNA isolated from SU-1, JW12, and JW13 at 24, 40, and 60 h. Densitometric analysis of agarose gels on which we resolved the full-length *atfB* transcript showed an approximately threefold increase in total *atfB* transcript levels in AtfB-silenced strains when compared with SU-1 at 24 h (Figure 1B). The total amount of *atfB* transcript produced in the AtfB-silenced strains represents the sum of the transcripts expressed at the native locus (n), plus transgene transcripts expressed from the ectopic site (e).

We then analyzed the ratio of native *atfB* transcript (n) to total *atfB* transcript (native *atfB* transcript, n + transgene *atfB* transcript, e) as well as the ratio of the transgene *atfB* transcript (e) to the total *atfB* transcript (n + e) expressed in AtfB-silenced strains at 24 h, 40 h, and 60 h by conducting a *Hind*III-cutting assay (see Methods). We amplified a 957 bp cDNA fragment in SU-1, JW12, and JW13 at three time points. This fragment was gel-excised and digested with *Hind*III. The presence of a *Hind*III-site inserted in the silencing construct enabled the subsequent analysis of the relative quantity of native (wild-type, n) and ectopic (transgene, e) transcript as detected by digestion with *Hind*III. The data demonstrated that the transgene *atfB* transcript is not expressed in SU-1, but is expressed in AtfB-silenced strains (Figure S3). When we calculated the transcript ratios for native (n/n + e) and transgene (e/n + e) *atfB* transcripts, we observed that JW12 and JW13 exhibit different patterns of expression of the silencing transcript. The relative quantity of transgene transcript (e/n + e) was highest at 24 h in JW12 (Figure 1B, bottom left panel), and this ratio decreased markedly at 40 h and 60 h. In contrast, constitutive and increasing levels of expression of the transgene transcript in JW13 maintained lower levels of native transcripts at 24 h, 40 h, and 60 h, with the highest relative quantity of silencing transcript (e/n) expressed at 60 h (Figure 1B, bottom right panel).

### 2.2. AtfB Directly Regulates Aflatoxin Biosynthesis and Fungal Development

**Figure 2:**
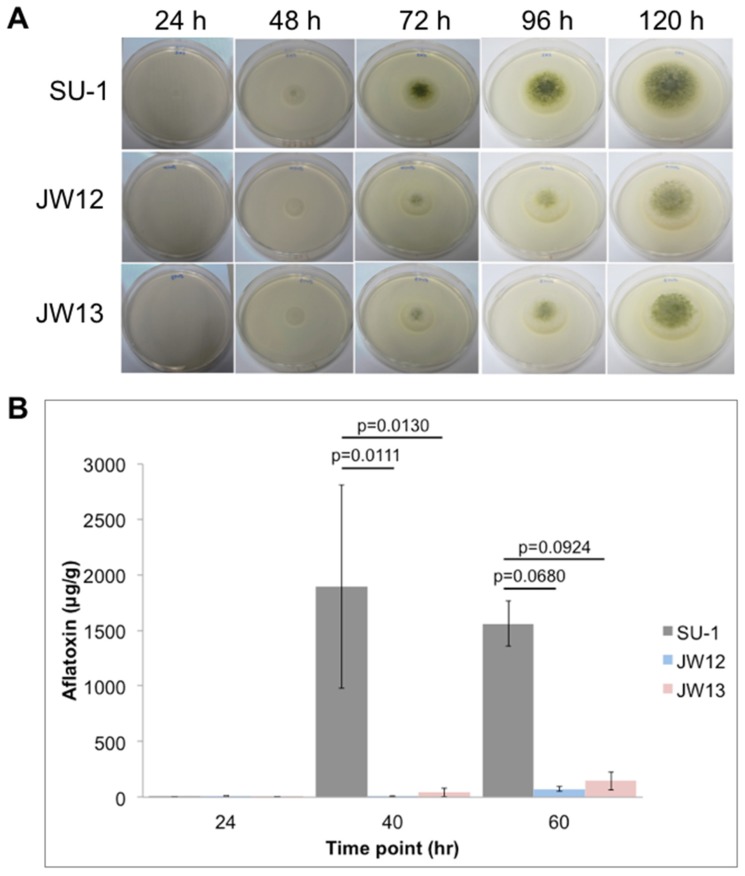
AtfB regulates secondary metabolism and development in *A. parasiticus*. Characterization of fungal growth, conidiospore number/pigment, and aflatoxin production in wild-type SU-1 and AtfB-silenced strains. (**A**) *A. parasiticus* strains were grown for 24 h, 48 h, 72 h, 96 h, and 120 h on aflatoxin-inducing solid potato dextrose agar (PDA), and analyzed for growth and conidiospore number/pigment. (**B**) *A. parasiticus* strains were grown for 24 h, 40 h, and 60 h in aflatoxin-inducing YES liquid media, and analyzed for aflatoxin production (ug) by ELISA normalized to mycelia dry weights (g). Aflatoxin levels (ug/g) are presented as the means ± SEMs of triplicates from one representative biological experiment (two independent biological experiments were conducted showing the same trend of aflatoxin production in AtfB-silenced strains). Error bars represent standard deviations. Data were analyzed for statistical significance using one-way ANOVA and a *post hoc* Tukey’s test for pairwise comparisons. *p* = 0.0111, SU-1 control 40 h compared with JW12 at 40 h; *p* = 0.0130, SU-1 control 40 h compared with JW13 at the same time point; *p* = 0.0680, SU-1 control 60 h compared with JW12 at 60 h; *p* = 0.0924, SU-1 control 60 h compared with JW13 at the same time point.

On an aflatoxin-inducing solid medium (potato dextrose agar, or PDA), the growth of AtfB-silenced strains and wild-type SU-1 was observed for 24 h to 120 h (for a total of five days). Silencing of AtfB at the genetic level resulted in impaired fungal development characterized by decreased conidiospore formation or spore pigmentation from 48 h to 120 h; however, the fungal growth rate was not significantly affected (Figure 2A).

We previously demonstrated that under standard aflatoxin-inducing conditions, *A. parasiticus* initiates aflatoxin synthesis between 24 h and 30 h, and maximum levels of synthesis occurs by 40 h [18,19]. In the current work, AtfB silencing results in a significant decrease of aflatoxin biosynthesis in AtfB-silenced strains as compared with the wild type. JW12 produced 200-fold less aflatoxin at 40 h and 40-fold less at 60 h, whereas JW13 produced 20-fold less aflatoxin at 40 h, and 10-fold less at 60 h when compared with SU-1 (Figure 2B and Figure S4). Taken together, these data support a direct association between AtfB silencing, aflatoxin synthesis, and fungal development.

#### 2.2.1. AtfB Directly Regulates Genes in the Aflatoxin Cluster

**Figure 3:**
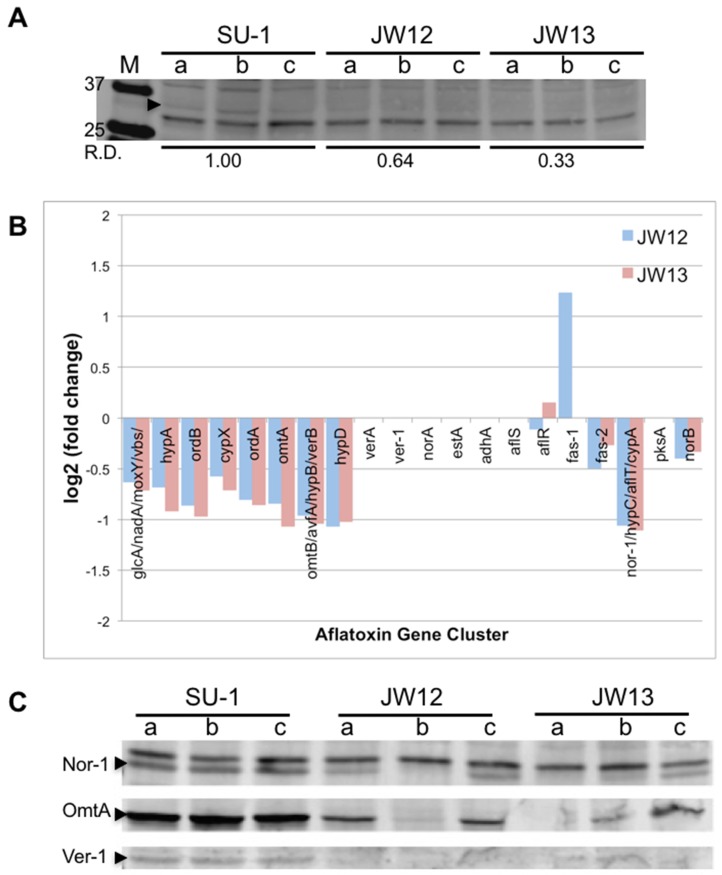
Decrease of the AtfB protein likely results in inability of AtfB to bind promoters of sentinel genes in aflatoxin biosynthesis. (**A**) AtfB protein levels analyzed by Western blot analysis. Strains SU-1, JW12, and JW13 were grown for 40 h on YES liquid medium, and proteins were extracted for Western blot analysis (see Methods). *A. parasiticus* proteins were detected on membranes using a highly specific anti-AtfB polyclonal antibody. 100 μg of total protein was loaded per lane. Arrow > indicates the location of the 34-kDa AtfB protein band. The molecular weight of AtfB was confirmed in a previous study [2] and by an AtfB immuoprecipitation and peptide competition analysis (see Figure S5). R.D., relative density measured by ImageJ densitometry analysis. (**B**) Expression analysis of gene targets in the aflatoxin cluster by RNA Seq analysis in AtfB-silenced strains. Blue bar graphs represent differentially expressed (DE) genes in JW-12 compared with SU-1, and red bar graphs (DE) genes in JW-13 compared with SU-1. Differential expression was calculated and reported as log base 2 of the fold change in expression. Bars that extend above zero on the y-axis are genes that showed up-regulation in expression as compared with SU-1. Bars that extend below zero on the y-axis are genes that were down-regulated. Common gene name(s) presented in the bar graphs (x-axis) are based on the annotation of the *A. flavus* aflatoxin gene cluster (reference genome). (**C**) Aflatoxin protein accumulation analyzed by Western blot analysis. Western blot analysis of aflatoxin enzymes Nor-1, OmtA, and Ver-1 accumulated in SU-1 and AtfB-silenced strains were grown in YES liquid medium for 40 h. A total of 10^6^ spores were inoculated into 100 mL YES liquid medium under standard conditions, and total proteins were extracted (see Methods). Protein extracts were prepared from triplicate samples for each strain.

We analyzed expression of the AtfB protein in SU-1 and AtfB-silenced strains using AtfB-specific anti-peptide polyclonal antibodies generated previously [2]. Anti-AtfB detected a 34-kDa protein in cell extracts of SU-1, JW-12, and JW-13 at 40 h, which was consistent with the previously reported mass for the AtfB protein [2], and supported by an AtfB immunoprecipitation and peptide competition analysis (Figure S5). Densitometry analysis demonstrate that the 34-kDa protein was detected at markedly decreased levels in AtfB-silenced strains at 40 h of growth under aflatoxin-inducing conditions as compared with SU-1 (Figure 3A).

At 40 h of growth in aflatoxin-inducing YES medium, we also observed an overall trend of down-regulation in the aflatoxin cluster. These transcripts are expressed at a minimum of twofold lower levels in atfB-silenced strains as compared with wild-type strain SU-1, although the observed differences in expression were not statistically significant (q > 0.05). These genes include *glcA*, *nadA*, *moxY*, *vbs*, *hypA*, *ordB*, *cypX*, *ordA*, *omtA*, *omtB*, *avfA*, *hypB*, *verB*, *hypD*, *fas-2*, *nor-1*, *hypC*, *aflT*, *cypA*, and *norB* (Figure 3B). In contrast, *fas-1* was up-regulated in JW12, but the transcript was not detected in JW13. Expression of *aflR* was down-regulated in JW12 and up-regulated in JW13; these changes in expression were at least twofold, but not statistically significant.

Next, we investigated whether the observed trend of the down-regulation of the aflatoxin gene cluster transcripts resulted in decreased levels of aflatoxin structural enzymes. We analyzed levels of the aflatoxin enzymes Nor-1, OmtA, and Ver-1 at 40 h of growth in aflatoxin-inducing medium by Western blot analysis using antibodies specific to Nor-1, OmtA, and Ver-1. Consistent with marked decreases of aflatoxin production (Figure 2B) and the overall trend of down-regulation of the aflatoxin cluster by RNA Seq, we observed lower levels of Nor-1, OmtA, and Ver-1 aflatoxin structural enzymes in both AtfB-silenced strains compared with SU-1 (Figure 3C). We conclude that the pattern of aflatoxin enzyme accumulation was closely associated with the pattern of AtfB protein accumulation as a result of AtfB silencing.

#### 2.2.2. AtfB Regulates Expression of Sentinel Virulence-Associated Genes

**Figure 4:**
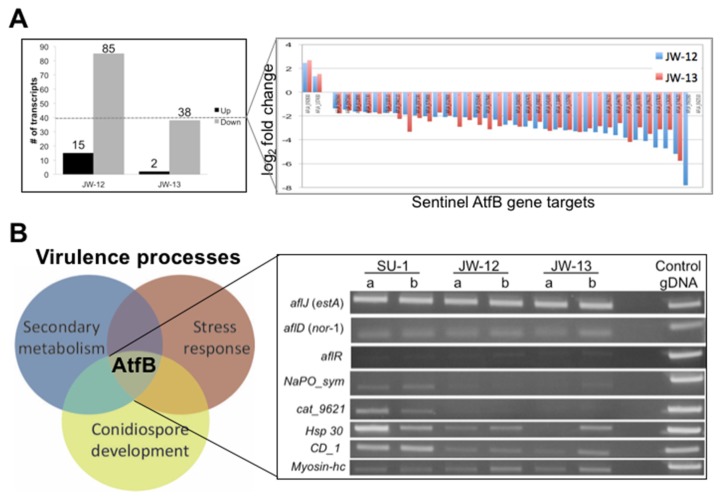
AtfB regulates expression of sentinel virulence-associated genes. Strains SU-1, JW-12, and JW-13 were grown for 40 h in YES liquid medium, RNA extracted, and then RNA Seq analysis was performed on duplicate samples for each strain (see Methods). (**A**) Transcripts that were differentially expressed (DE) between the AtfB-silenced strains JW-12 and JW-13 compared with SU-1 are shown (*q*-value < 0.05). (Left panel) The number of transcripts that are up-regulated (black bars) or down-regulated (grey bars) are indicated by different colored bars. (Right panel) Forty overlapping differentially expressed genes were identified in AtfB-silenced strains compared with SU-1. Blue bars represent JW-12, and red bars represent JW-13. DE was calculated and reported as log base 2 of the fold change in expression (y-axis). AFLA_gene designation is presented on the x-axis. (**B**) RT-PCR validation of eight representative genes from RNA Seq analysis. Expression of three aflatoxin genes (aflD, aflJ, aflR) and five other genes encoding a sodium/phosphate symporter (NaPO_sym), a putative catalase (cat_9621), a heat shock protein (Hsp 30), a conidiation family protein (CD_1), and a heavy chain myosin-related gene, as detected by RT-PCR (right panel). Genomic DNA (50 ng) was used as a control. Based on the expression patterns, identity, and function of selected sentinel genes, we show that AtfB functionally links at least three virulence-associated cellular processes (secondary metabolism [SM], stress response [SR], and conidiospore development [CD]) (left panel).

As part of the RNA Seq analysis, we were interested in identifying specific target genes regulated by AtfB (Figure 4). We collectively designated this group of genes as “sentinel genes”, because AtfB silencing is associated with their down-regulation. We observed that 100 transcripts in JW12 and 40 transcripts in JW13 were differentially expressed at statistically significant (q < 0.05) levels when compared with wild-type SU-1 during growth under aflatoxin-inducing conditions. All 40 of the transcripts differentially expressed in JW13 were also differentially expressed in JW12. We focused further efforts on the 40 overlapping, differentially expressed (DE) genes that were identified in both AtfB-silenced strains. Thirty-eight of these 40 gene targets were down-regulated, while two genes (AFLA_092830, which encodes a putative Rho-guanyl nucleotide exchange factor, and AFLA_107430, which encodes a hypothetical protein) were up-regulated (Figure 4A; Dataset S2).

We utilized RT-PCR analysis to validate the differential expression of eight candidate genes selected from 40 overlapping genes identified by RNA Seq analysis (Figure 4B). The four genes (AFLA_074620 encodes a sodium/phosphate symporter, AFLA_096210 encodes a putative catalase, AFLA_037820 encodes a Hsp-30-like heat shock protein, AFLA_044790 encodes a conidiation-specific family protein) with the highest levels of differential expression in AtfB-silenced strains were confirmed by RT-PCR analysis to be down-regulated in support of RNA Seq analysis. In contrast, RT-PCR analysis did not detect differences in the expression of the four genes (the aflatoxin genes *aflJ*, *aflD*, and *aflR*, and AFLA_062510, which encodes a heavy chain myosin protein).

#### 2.2.3. The AtfB Regulatory Network Extends beyond Canonical Oxidative Stress Response Pathways Associated with Fungal bZIPs

**Figure 5:**
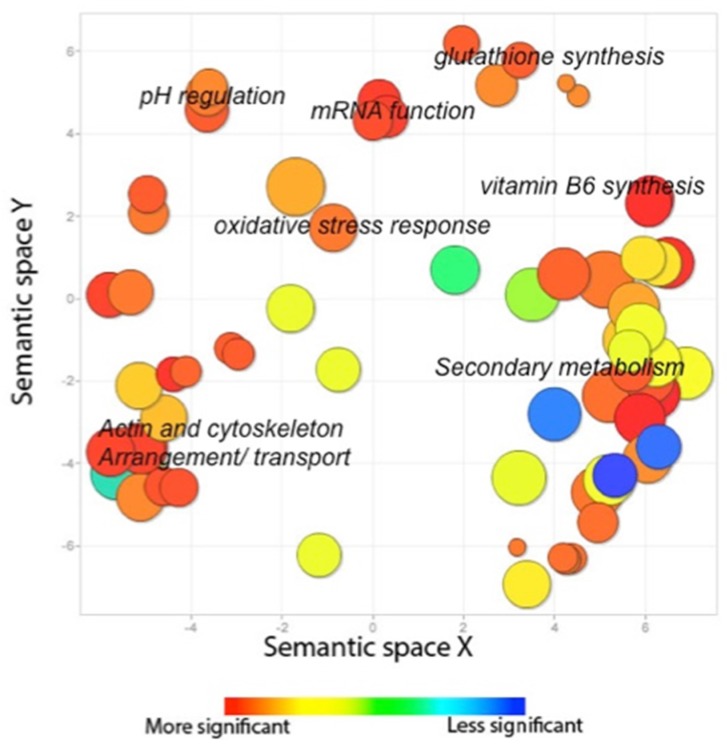
The fungal AtfB regulatory network extends beyond canonical oxidative stress response pathways. Strains SU-1, JW-12, and JW-13 were grown for 40 h in aflatoxin-inducing YES liquid medium, RNA was extracted, and RNA Seq analysis was performed on duplicate samples for each strain (see Methods). RNA Seq analysis of the entire transcriptome was subjected to pathway enrichment analysis to assess the functional significance of altered AtfB function in AtfB-silenced strains compared with wild-type SU-1. Gene ontology (GO) terms represented by circles are visualized in a semantic similarity-based scatter plot, where similar GO terms with *p*-values < 0.05 are in close proximity to each other as determined by REViGO (see Methods). The area of the circle is proportional to the significance of the over-representation of the GO term (-log base10 *p*-value). The color of the circles represents the statistical significance of the over-representation of the GO term as shown in the legend: red indicates a higher degree of functional significance compared with blue, which indicates a lower degree of functional significance. For GO term annotations, see Supplemental Table S3.

To obtain a comprehensive picture of the network of signaling pathways that regulate oxidative stress response in *A. parasiticus*, the entire transcriptome (approximately 14,000 gene targets) obtained from RNA Seq of AtfB-silenced strains was annotated based on the *Aspergillus fumigatus* gene ontology (GO) database. *A. fumigatus* is an important human pathogen, and the ability to cope with oxidative stress enhances the disease capability of *A. fumigatus* in host tissue [20]. To assess the potential relevance of oxidative stress pathways in *A. parasiticus* and *A. fumigatus*, we compared the expression of genes in AtfB-silenced strains to expression in the wild-type strain SU-1 using pathway enrichment analysis. To simplify the visualization of GO terms and enriched biological pathways that were altered in AtfB-silenced strains, REVIGO web-based software was used, based on the idea that similar ontology terms with similar biological functions are clustered together. This clustering is represented in semantics space or biological meanings (Figure 5, also see Figures S6 and S7). Biological processes in “secondary metabolism” was the most over-represented GO term, followed by “actin cytoskeleton rearrangement and transport”. Other over-represented pathways in AtfB-silenced strains were gene targets related to “oxidative stress response”, “pH regulation”, “mRNA function”, “glutathione synthesis”, and “vitamin B6 synthesis”.

## 3. Discussion

### 3.1. Functional Significance of the AtfB Regulatory Network

In recent years, many authors reported evidence of a close association between secondary metabolism, oxidative stress, and development in fungi [21,22] . Although this association exists, why these cellular processes are intricately connected is still unknown. Fungal transcription factors that belong to the bZIP family are historically known for their ability to mediate stress responses triggered by developmental or environmental signals [13,23]. In particular, adaptation to oxidative stress is postulated to be an important virulence-associated cellular process. The ability to cope with stress enhances virulence and increases the ability of fungi to establish infection in humans, plants, and animals. *Aspergillus* bZIP proteins including AP-1, AtfA, AtfB, FlbB, JlbA, MeaB, and RsmA are reported to be associated with one or more cellular networks involved in stress response, secondary metabolism, and conidiospore development [4,5,6,9,10,11,20]. In particular, AtfA and AtfB belong to the Atf/CREB group of transcription factors that can form hetero- or homodimers and bind to the CRE sequences (T[G/T]ACGT[C/A]A) found in promoters of oxidative and osmotic stress response genes [6]. Due to high DNA-binding domain sequence similarity, AtfA and AtfB are likely to control a similar subset of genes containing CRE motifs and regulate specific cellular functions in response to environmental stress. A well-known example of such cellular function is the ability of *A. oryzae* AtfA and AtfB to control conidial tolerance and resistance to stress [4,6]. Although there is speculation that these virulence-associated processes are co-regulated by specific key players, the extent of this regulatory network remains unclear.

One way to study the rewiring of a transcriptional network in response to environmental cues is to focus on a single biological function. So, we used secondary metabolism in the model organism, *A. parasiticus*, in order to pursue this objective. In *A. parasiticus*, our previous studies [2,14,15] demonstrated that AtfB binds to promoters of aflatoxin and stress response genes containing the CRE consensus sequence, 5′-T(G/T)ACGT(C/A)A-3′, which that we call CRE1. The ability of AtfB to physically interact with the aflatoxin pathway regulator AflR, and bind CRE sites in promoters of secondary metabolism and stress response genes prompted the idea that this particular transcription factor could play a direct role in the synthesis of aflatoxin and other secondary metabolites. Due to the physical location of *aflR* within the aflatoxin cluster, much effort has been focused on the direct role of AflR in the regulation of a specific subset of genes involved in aflatoxin biosynthesis. Previous studies suggest that AflR regulates genes outside the aflatoxin cluster, and the number of genes regulated by AflR is likely larger than previously identified. In support of this, 3647 putative AflR-binding sites were identified in the *A. flavus* genome [17].

Computer-based Multiple Em for Motif Elicitation (MEME) analyses of consensus sequences in AtfB sentinel genes identified through RNA Seq analysis (conducted in the current study) and ChIP Seq analysis (conducted previously) strongly support the role of AtfB binding to promoters beyond core stress-related genes. Putative AtfB sites were identified in three out of four sentinel genes (AFLA_044800, conidiation specific protein (three sites); AFLA_037830, heat shock protein 30 (three sites); AFLA_074630, sodium/phosphate symporter (1 site)), which provides critical evidence that supports the direct regulation of these promoters by AtfB (see Supplemental Material Figure S6). Furthermore, the location and position of the CRE site is in close proximity with the AflR-binding site, as well as to Forkhead box class O (FOXO)-like transcription factor binding sites. Of particular importance for further analysis, the promoter region of a sodium/phosphate symporter (AFLA_074630; one of the AtfB sentinel genes identified above) contains consensus sequences for all three sites: CRE-like motifs, AflR-binding motifs, and a FOXO-like motif. Based on these observations, we speculate that AtfB plays both a direct and an indirect role in the regulation of sentinel genes dependent on the presence and location of CRE sites, AflR sites, or both. We hypothesize that AtfB directly regulates the expression of sentinel genes by binding to CRE motifs, and indirectly regulates these and other genes by recruiting binuclear zinc cluster transcription factors (C6 TF) or FOXO-like transcription factors to specific promoters.

We observed that AtfB silencing is strongly associated with a specific phenotype characterized by white fluffy mycelium, low conidiospore numbers and pigment, and low aflatoxin levels. These data support observations that link fungal development and secondary metabolism [13,22,24,25,26]. For example, the loss of AtfA or AtfB function in *A. oryzae* was demonstrated to impair conidial development (white fluffy mycelium phenotype), and was associated with increased sensitivity to oxidative stress [4,6]. In further support of this phenotype, various conidiation mutants reported in a previous study were designated with “fan” and “fluff” phenotypes, because they were characterized by altered conidiospore development and a reduction in aflatoxin production [27,28]. Moreover, the deletion of a light-sensitive regulator, VeA in *A. parasiticus*, results in reduced conidiation, the blockage of sclerotial formation (resistant survival structures), and non-detectable levels of aflatoxin production [29]. We proposed that the phenomemon of epistasis (i.e., which gene occurs first in a biochemical or regulatory pathway) [30,31] likely contributes to phenotypic variation in conidiospore development and secondary metabolite production.

Part of the future focus of work in our laboratory will further explore the implications of the key network evidence that AtfB regulates actin assembly in the cell (Figure 5). This novel extension to the AtfB regulatory network is in line with our model and parallel findings, in which specific transport vesicles (toxisomes) carry structural enzymes and pathway intermediates, fuse to form endosomes, and then store and/or export secondary metabolites to the cell exterior [19,24,32,33,34,35]. We propose that gene targets involved in this intracellular transport machinery likely play a role in facilitating the subcellular localization of aflatoxin enzymes and the export of aflatoxin. AtfB silencing negatively regulates the export pathway (by decreasing synthesis of aflatoxin), thus down-regulating gene targets critical to aid in the transport of aflatoxisomes to fuse to the cell membrane. Functional actin filaments are critical for mitochondrial morphology and the dynamic behavior of the mitochondrial network in *A. nidulans* [36]. Mitochondria are essential organelles for oxidative energy metabolism and an important source of intracellular reactive oxygen species (ROS); an exciting extension to this finding is to focus on the role of actin cytoskeleton in oxidative stress response and secondary metabolism.

### 3.2. Key Features of RNAi-Based Gene Silencing of AtfB

Two copies of AtfB were identified by high stringency Southern blot analysis in *A. parasiticus* and *A. flavus*. The AtfB-silencing strategy (Figure S1) was designed to knockout at least one copy of AtfB, thus enabling us to observe possible changes in phenotype in the initial knockout strain. We screened 93 resulting transformants in the first knockout stage through using a non-biased sib-selection/PCR approach to identify transformants that carried the knockout construct. The screen identified several “sibs” with at least one transformant that carried the construct, and the individual isolates within these “sibs” were later identified (including JW12 and JW13). Surprisingly, and to our disappointment, none of the transformants screened showed evidence of a gene knockout event. However, one in every three transformants carried the silencing construct, and most importantly, all of the transformants that carried the silencing construct exhibited a new phenotype characterized by reduced levels of conidia, conidial pigment, and aflatoxin (a white ‘fluffy’ mycelium phenotype) (Figure 4). All other transformants that lacked the silencing construct exhibited the wild-type phenotype (normal levels of conidia, conidial pigment, and aflatoxin). In support of the utility of this endogenous gene-silencing mechanism, we recently conducted gene silencing of *vps34*, a gene encoding a vacuolar-sorting protein using a similar co-transformation method [37]. In this study, high levels of transgene transcripts expressed by the Vps34 silencing construct at 40 h down-regulated expression of wild-type transcripts at that time point. The phenotype associated with Vps34 is opposite to that observed in AtfB. Vps34-silenced strains exhibit increased levels of conidiospores, increased levels of aflatoxin, and increased production of sclerotia. So, one major benefit of this approach is that it provides an effective means to rapidly test gene function, including gene targets that are potentially lethal in *Aspergillus* spp. by RNAi-based gene silencing or “quelling”.

“Quelling” was originally described in the ascomycete *Neurospora crassa* [38,39,40]. Considering that *A. parasiticus* also belongs to the same genus as *N. crassa* (Ascomycota), it is not surprising that a similar silencing mechanism exists in both *N. crassa* and *A. parasiticus*. Key features of this silencing mechanism observed in both organisms include: multiple copies of the transgene integrate in tandem at ectopic sites; the native (wild-type) gene copy is not replaced by the gene knockout construct; the transgene is expressed at the transcript level; silencing at the level of transcript down-regulates protein levels; and the presence and expression of the transgene is directly linked to the observed changes in phenotype [38,39,40]. Thus, the “quelling” phenomenon provides a relatively straightforward opportunity to quickly engineer industrially relevant *Aspergillus* strains such as *A. oryzae* (soy sauce production) and *A. niger* (enzyme production) to rapidly test the function of candidate genes involved in the fermentation and production of beneficial secondary metabolites. The presence and further characterization of this endogenous gene-silencing mechanism (“quelling”) in *Aspergillus* strains presents a promising avenue to down-regulate aflatoxin synthesis in host plants through transgenic plants producing the silencing cassette. In a recent study [41], maize plants that were transformed with a silencing construct targeting the transcription factor aflR demonstrated a 14-fold reduction of aflatoxin levels compared with wild-type plants. Due to low levels of *aflR* expression, the authors hypothesized that the silencing construct processed the *aflR* transcript into 21–28 bp fragments (via “quelling”), leading to downstream silencing of the aflatoxin cluster [41].

## 4. Conclusions

Our current work demonstrates that the fungal transcription factor AtfB regulates virulence-associated cellular processes involved in secondary metabolism, stress response, and fungal development. The silencing of AtfB results in a strong impact on the level of aflatoxin synthesis and impaired conidial development. Although canonically known as an oxidative stress-related transcription factor, this work suggests that AtfB is a master regulator of secondary metabolism, development, cellular defense, and intracellular transport machinery. Due to the low numbers of bZIP proteins in *Aspergillus* genomes, the AtfB gene regulatory network provides a large number of excellent cellular targets to control molecular mechanisms that contribute to fungal virulence.

## 5. Materials and Methods

### 5.1. Fungal Strains and Growth Conditions

*A. parasiticus* strain SU-1 (ATTC 56775) was the wild-type aflatoxin-producing strain used in this study. *A. parasiticus* NR-1, which harbors a non-functional gene encoding nitrate reductase (*niaD*), was derived from SU-1 and used as a recipient and control strain for co-transformation [42].

YES liquid medium (2% yeast extract and 6% sucrose, pH 5.8) was used as an aflatoxin-inducing growth medium. Conidiospores from frozen mycelia stock solutions were inoculated into 100-mL liquid medium at a final concentration of 10^4^ spores per mL; each flask contained five 6-mm glass beads (Sigma) [43]. Cultures were incubated at 30 °C with shaking at 150 rpm in the dark for designated time periods (standard conditions). Potato dextrose agar (PDA; Becton Dickinson, Franklin Lakes, NJ, USA) was used as an aflatoxin-inducing solid growth medium. 10^4^ spores (10 μL) were center-inoculated onto the solid medium surface, which was then incubated for 24 h to 120 h at 30 °C in the dark.

For RNA Seq analysis, duplicate samples of SU-1, JW-12, and JW-13 were grown in YES liquid medium for 40 h under standard growth conditions (30 °C with shaking at 150 rpm in the dark).

### 5.2. Creation of AtfB-Silenced Strains

#### 5.2.1. Silencing Constructs

AtfB was previously identified and designated AFLA_094010 (92.m03394) using the published *A. flavus* genome database (http://www.aspergillusflavus.org/) by strong sequence identity (96%) to the transcription factor AtfB in *A. oryzae* [2]. The *A. parasiticus* AtfB sequence identity was confirmed by cloning, nucleotide sequence analysis, and comparison against the recently published *A. parasiticus* genome sequence, which was determined by Illumina sequence analysis [2,44]. The nucleotide sequence accession number for *atfB* is ADZ06147.1, and the *A. parasiticus* SU-1 genome sequence is available through the NCBI database (Accession: JMUG00000000) (http://www.ncbi.nlm.nih.gov/).

We amplified two fragments (Fragment 1, 5′-ATGTCGGTGGACCAAACCCT-3′, forward primer, Fragment 2, 5′-GGCGAAGAAGCTTAGAACAGTTGCTCACTCTGGTCGACG-3′, reverse primer; Fragment 2, 5′- TGTTCTAAGCTTCTTCGCCGAAAAATTCCTAGAACGGAA-3′, forward primer, 5′- CTAAACATTAATCAGCTCTT-3′, reverse primer) from an intronless *atfB* ORF of 957 bp by PCR. The overlap extension PCR of Fragment 1 and 2 resulted in a 20-bp deletion and insertion of a *Hind*III-site in the silencing construct (Figure S1). Fifty nanograms of gDNA was used as a template under the following conditions: using both primer pairs to generate fragment 1 and 2, then a subsequent PCR using the forward primer from fragment 1, and the reverse primer of fragment 2 using both gel purified fragments as template. PCR conditions are as follows: initial denaturation at 94 °C for 4 min; followed by 35 cycles of 94 °C for 1 min, 60 °C for 2 min, and 72 °C for 1 min, with a final extension at 72 °C for 9 min.

#### 5.2.2. Fungal Transformation and Strain Confirmation

A circular and uncut plasmid pSL82 containing a 6.3-kb *Hind*III fragment with the selectable marker *niaD* was used together with the silencing construct for the co-transformation of strain NR-1 [42]. Protoplasts were obtained as previously described [42,45] from strain NR-1 (*niaD*–, aflatoxin+). A “sibling” (sib) selection method was used to screen resulting transformants. PCR and Southern blot analyses of *niaD* + transformants identified several in which multiple tandem copies of the silencing construct integrated into the *A. parasiticus* genome at ectopic sites. The AtfB-silenced strains JW12 and JW13 were selected based on PCR and Southern blot analysis for further characterization. We define JW12 and JW13 as “silenced strains” based on *atfB* transcript and AtfB protein levels observed.

### 5.3. DNA Extraction and Southern Blot Analysis

Genomic DNA from *A. parasiticus* NR-1, JW12, and JW13 was isolated from frozen fungal mycelium using the phenol-chloroform method previously described [45]. Southern hybridization analysis was performed as previously described [46] with minor modifications. Briefly, 2.5 μg genomic DNA was digested with 20 U of *Bam*HI-HF, *Eco*RI-HF, or *Hind*III at 37 °C for 16 h (NEB, Ipswich, MA, USA). Digested DNA was separated on a 0.8% agarose gel and transferred onto a Nytran^®^ SuPerCharge membrane (Schleicher and Schell, Inc., Keene, NH, USA) by capillary transfer. Membranes were UV cross-linked (120,000 μJ /cm^2^) in a UV Stratalinker^®^ 2400 (Stratagene, Inc., La Jolla, CA, USA). A 600-bp *atfB* probe was generated by PCR (5′-ATGTCGGTGGACCAAACCCT-3′, forward primer; 5′-TCGCCTTTCTTGCTGGATAC-3′, reverse primer) (Figure S1). PCR products were purified with a Qiagen Gel Purification kit (Qiagen, Valencia, CA, USA). Probe labeling, hybridization, the washing of membranes at 55 °C, and detection were performed using an Amersham Gene Images AlkPhos Direct Labeling and Detection System (GE Healthcare, Pittsburg, PA, USA) according to manufacturer’s instructions.

### 5.4. Aflatoxin Measurements in SU-1 and AtfB-Silenced Strains

Triplicate cultures of SU-1, JW12, and JW13 were grown in YES liquid medium under standard conditions (30 °C with shaking at 150 rpm in the dark) and harvested at designated time points. Aflatoxin B_1_ concentration (μg) in the medium was quantified by ELISA using anti-aflatoxin B_1_ polyclonal antibodies (Sigma-Aldrich, St. Louis, MO, USA) and normalized to mycelial dry weight (g), as previously described [46,47]. Data were analyzed for statistical significance using the computing environment R (R Development Core Team, 2005) using one-way ANOVA and a post hoc Tukey’s test for pairwise comparisons.

### 5.5. RNA Isolation, Transcript Analysis, and HindIII Cutting Assay

Triplicate cultures of SU-1, JW12, and JW13 were grown in YES liquid medium under standard conditions, harvested at 24 h, 40 h, and 60 h, respectively, and fungal cells were disrupted using a mortar and pestle in the presence of liquid nitrogen. TRIzol^®^ reagent (Invitrogen, Grand Island, NY, USA) was used to extract total RNA from ground mycelium [2]. An Agilent 2100 Bioanalyzer (Agilent Technologies, Santa Clara, CA, USA) was used to assess RNA quality. Total RNA (1 μg) was reverse-transcribed into cDNA with the QuantiTect reverse transcription kit (Qiagen).

A full-length *atfB* transcript was amplified with *atfB* specific primers (5′-ATGTCGGTGGACCAAACCCT-3′, forward primer; 5′-CTAAACATTAATCAGCTCTT-3′, reverse primer) by semi-quantitative PCR of cDNA prepared from cultures after 24 h, 40 h, and 60 h of growth. A 1-kb *atfB* fragment was purified using a Qiagen Gel Purification System. An equal volume of purified 1-kb *atfB* fragment was digested with 20 U of *Hind*III for 1 h, or left undigested to serve as a control. Band intensities and the ratio of wild-type to transgene transcripts were quantified using ImageJ [48].

### 5.6. Protein Extraction and Western Blot Analysis

Triplicate cultures of SU-1, JW12, and JW13 were grown in YES liquid medium for 40 h under standard conditions, harvested, and disrupted with a mortar and pestle under liquid nitrogen, and a protein extract was prepared as previously described [2]. Total protein concentrations were measured using the Pierce^®^ BCA Protein Assay Reagent (Thermo Scientific, Waltham, MA, USA). 100 μg of total proteins per lane was separated by SDS-PAGE using a 4–20% gradient Tris-HCl Ready Gel^®^ (Bio-Rad, Hercules, CA, USA) and transferred to polyvinylidene difluoride (PVDF) membranes (PerkinElmer Life Sciences, Waltham, MA, USA). Membranes were exposed to polyclonal anti-AtfB antibodies (YSR, 5 μg/mL) and incubated with goat anti-rabbit secondary antibodies conjugated to the fluorescent tag IRDye 800 CW (Li-Cor Biosciences, Lincoln, NE, USA) as previously described [2]. For aflatoxin enzymes, highly specific antibodies against Nor-1, OmtA, and Ver-1 were used as previously described [46,49,50,51]. Visualization of proteins was performed using an Odyssey infrared imaging system (Li-Cor Biosciences) at 795 nm, as previously described [2].

### 5.7. RNA Isolation and cDNA Library Preparation for RNA Seq Analysis

Duplicate cultures of SU-1, JW12, and JW13 were grown in YES liquid medium under standard conditions, harvested after 40 h, and ground in a mortar and pestle under liquid nitrogen, and RNA was extracted as described above. Library construction and RNA quality analysis for all six isolates were conducted by Otogenetics (Norcross, GA, USA) and Functional Biosciences (Madison, WI, USA). Integrity and purity of the RNA samples were assessed using an Agilent 2200 TapeStation Bioanalyzer System (Agilent, Santa Clara, CA, USA). All samples had an RNA Integrity Number (RIN) of 9.0 or higher with an OD260/280 between 1.78–1.80.

cDNA library construction was conducted as follows: Fragment AnalyzerTM (AATI, Ames, IA, USA) and Qubit^®^ were used to assess cDNA quality. 100 ng of total RNA was reverse transcribed into cDNA using a Clontech SmartPCR cDNA kit (Clontech, Mountain View, CA, USA). Restriction enzyme digestion end repair was conducted by removing adaptor sequences, and fragmentation of the resulting cDNA was conducted using a Covaris M220 focused-ultrasonicator (Woburn, MA, USA). Fragmented cDNA was subjected to Illumina library preparation with NEBNext-based reagents (NEB, Ipswich, MA, USA). The quality, quantity, and size distribution of Illumina libraries were determined using an Agilent Bioanalyzer 2100. cDNA clusters were generated on a cBot automated station, and the Illumina libraries were submitted for Illumina HiSeq2000 2 × 100 bp paired-end sequencing (Illumina, San Diego, CA, USA).

### 5.8. RNA Seq Analysis

RNA Seq data analysis was performed by ContigExpress, LLC (New York, NY, USA). Six *A. parasiticus* samples (duplicates of SU-1, JW12, and JW13) prepared as described above were used in this analysis. Detailed methods for QC of Ilumina sequence reads, mapping, annotation, and differential expression analysis were recently published [44]. Scatter plots with Spearman’s correlation (R^2^ > 0.80) demonstrates good agreement between technical replicates of RNA Seq data from all strains (Figure S8).

### 5.9. RT-PCR Analysis

Semi-quantitative PCR analyses was used to validate differential gene expression analysis obtained from RNA Seq. Total RNA (1 μg) was reverse-transcribed into cDNA with the QuantiTect reverse transcription kit (Qiagen), according to the manufacturer’s instructions. 1 μL (50 ng) of cDNA was used as a template in the subsequent PCR using a Robocycler Gradient 96 (Stratagene, La Jolla, CA, USA) under the following conditions: initial denaturation at 94 °C for 4 min; followed by 30 cycles of 94 °C for 1 min, 60 °C for 2 min, and 72 °C for 1 min, with a final extension at 72 °C for 9 min. Thirty cycles was chosen after primer–template optimization at 25, 30, and 35 cycles. The PCR products were separated by electrophoresis on a 0.8% agarose gel.

### 5.10. Gene Ontology (GO) Pathway Analysis and REVIGO

GO terms and pathway analysis was conducted by ContigExpress, LLC (New York, NY, USA). The entire transcriptome (approximately 14,000 transcripts) of JW12 and JW13 was annotated against the *A. fumigatus* database using KOBAS 2.0 [52]. About 80% of genes were annotated in at least one GO term or functional pathway. Differentially expressed genes in JW12 vs. SU1 and JW-13 vs. SU1 were subject to GO terms and pathway enrichment analysis using KOBAS 2.0. Enriched GO terms with *p*-values < 0.05 were used as an input list for REVIGO analysis (free software available at http://revigo.irb.hr/) [53].

## Supplementary Materials

The following are available online at www.mdpi.com/2072-6651/9/9/287/s1, Figure S1: Gene silencing of *A. parasiticus* AtfB, Figure S2: Genotypic screening of *atfB* transformants, Figure S3: PCR-based assay for validation of AtfB-silencing, Figure S4: Aflatoxin levels in JW12 and JW13 not detectable by TLC, Figure S5: AtfB immunoprecipitation and peptide competition analysis, Figure S6: Gene ontology visualization of JW12, Figure S7: Gene ontology visualization of JW13, Figure S8: Scatter plots with Spearman’s correlation (R^2^ > 0.80) showing good agreement between technical replicates of RNA Seq data from *A. parasiticus* wild-type strain, JW12, and JW13 grown under aflatoxin inducing YES conditions, Table S1: Primers used in this study, Dataset S1: Gene ontology (GO) enrichment analysis conducted on AtfB-silenced strains, JW12 and JW13, Dataset S2: Differentially expressed genes identified by RNA Seq analysis in AtfB-silenced strains, Dataset S3: Statiscally significant differential expression of AtfB sentinel genes identified by RNA Seq analysis in AtfB-silenced strains.
