# Supplementary material for: The Fungal bZIP Transcription Factor AtfB Controls Virulence-Associated Processes in Aspergillus parasiticus
Source: Toxins (Basel). 2017 Sep 16;9(9):287. doi: 10.3390/toxins9090287 (PMC5618220; doi:10.3390/toxins9090287)
Supplement: Supplementary file 1 [file toxins-09-00287-s001.zip › supple for 2 round review/toxins-218372 supple after 1 round.pptx]

## Slide 1
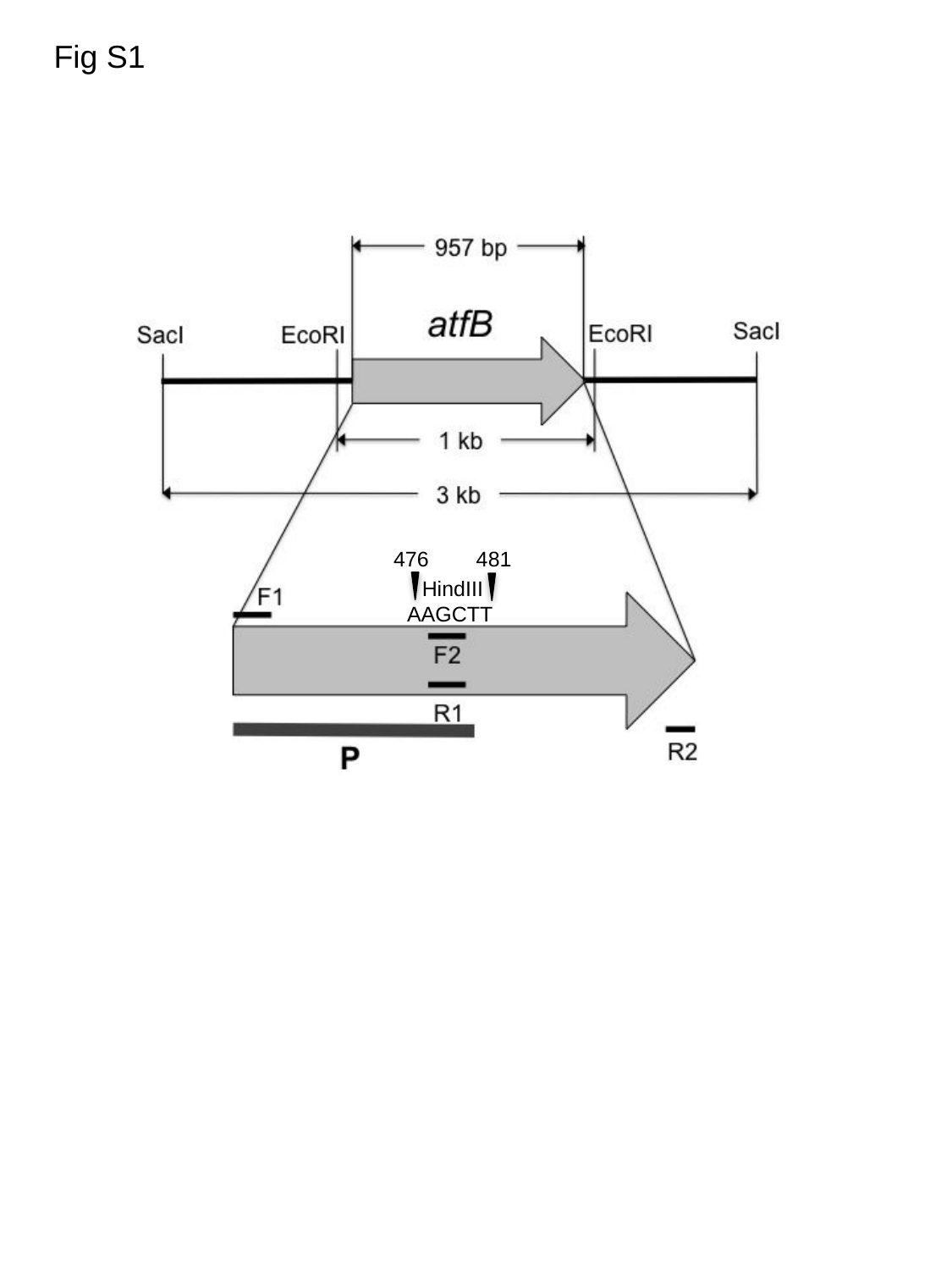

Fig S1
476 481
HindIII
AAGCTT

## Slide 2
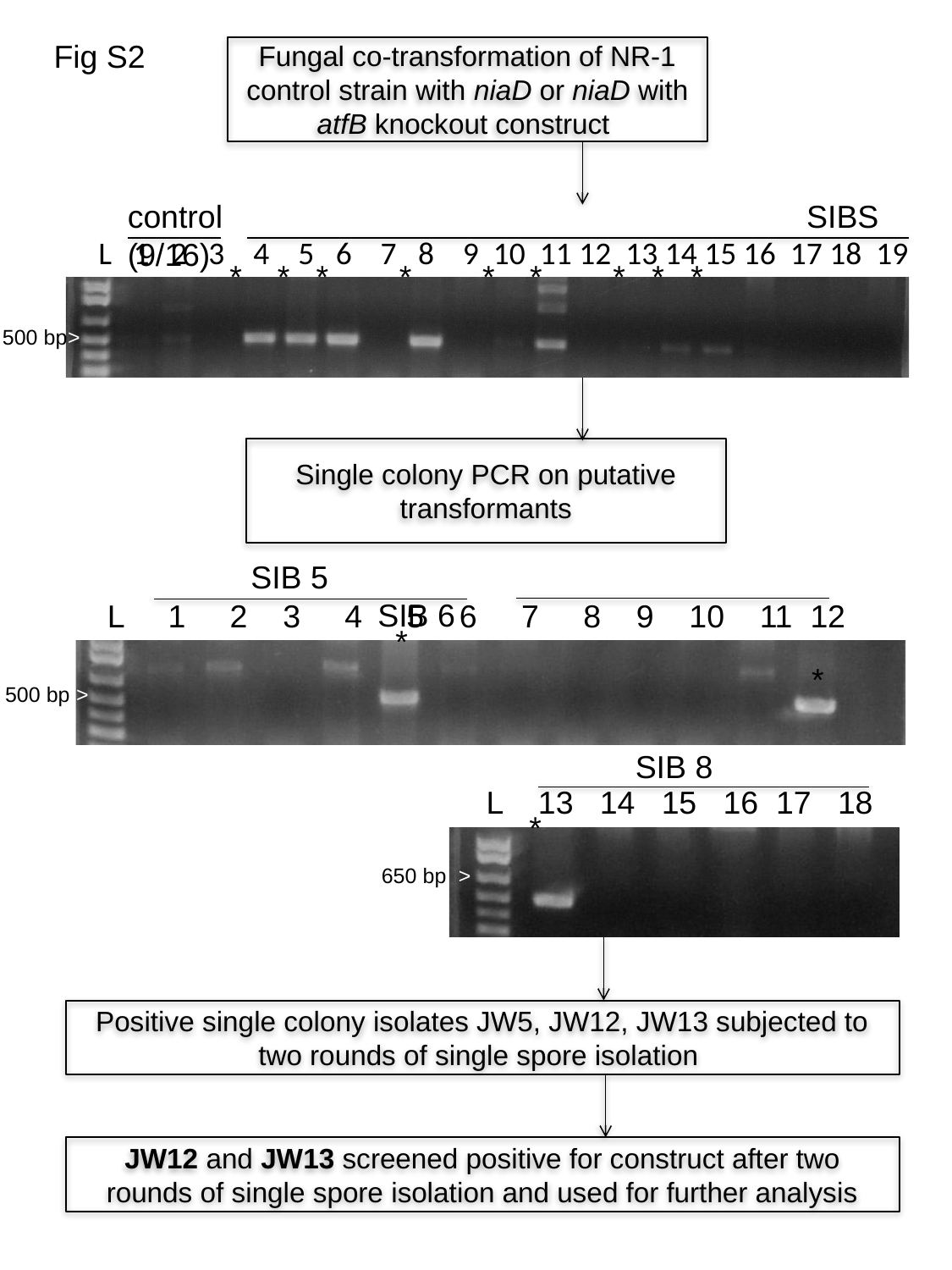

Fig S2
Fungal co-transformation of NR-1 control strain with niaD or niaD with atfB knockout construct
control					 SIBS (9/16)
L 1 2 3 4 5 6 7 8 9 10 11 12 13 14 15 16 17 18 19
* * * * * * * * *
500 bp>
Single colony PCR on putative transformants
SIB 5					SIB 6
L 1 2 3 4 5 6 7 8 9 10 11 12
*						 *
500 bp >
SIB 8
L 13 14 15 16 17 18
*
650 bp >
Positive single colony isolates JW5, JW12, JW13 subjected to two rounds of single spore isolation
JW12 and JW13 screened positive for construct after two rounds of single spore isolation and used for further analysis

## Slide 3
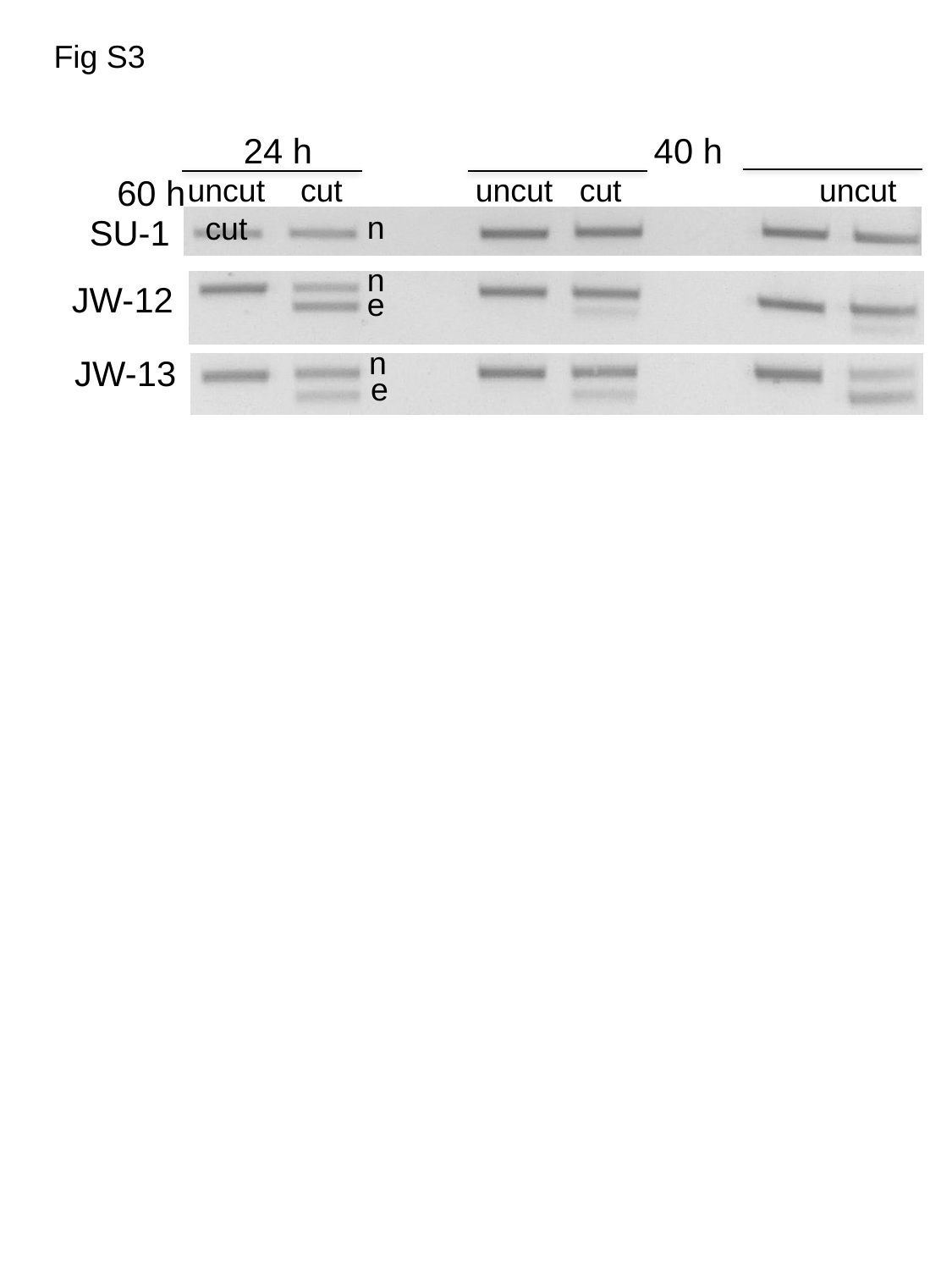

Fig S3
 24 h			 40 h 60 h
uncut cut uncut cut	 uncut cut
n
SU-1
n
JW-12
e
n
JW-13
e

## Slide 4
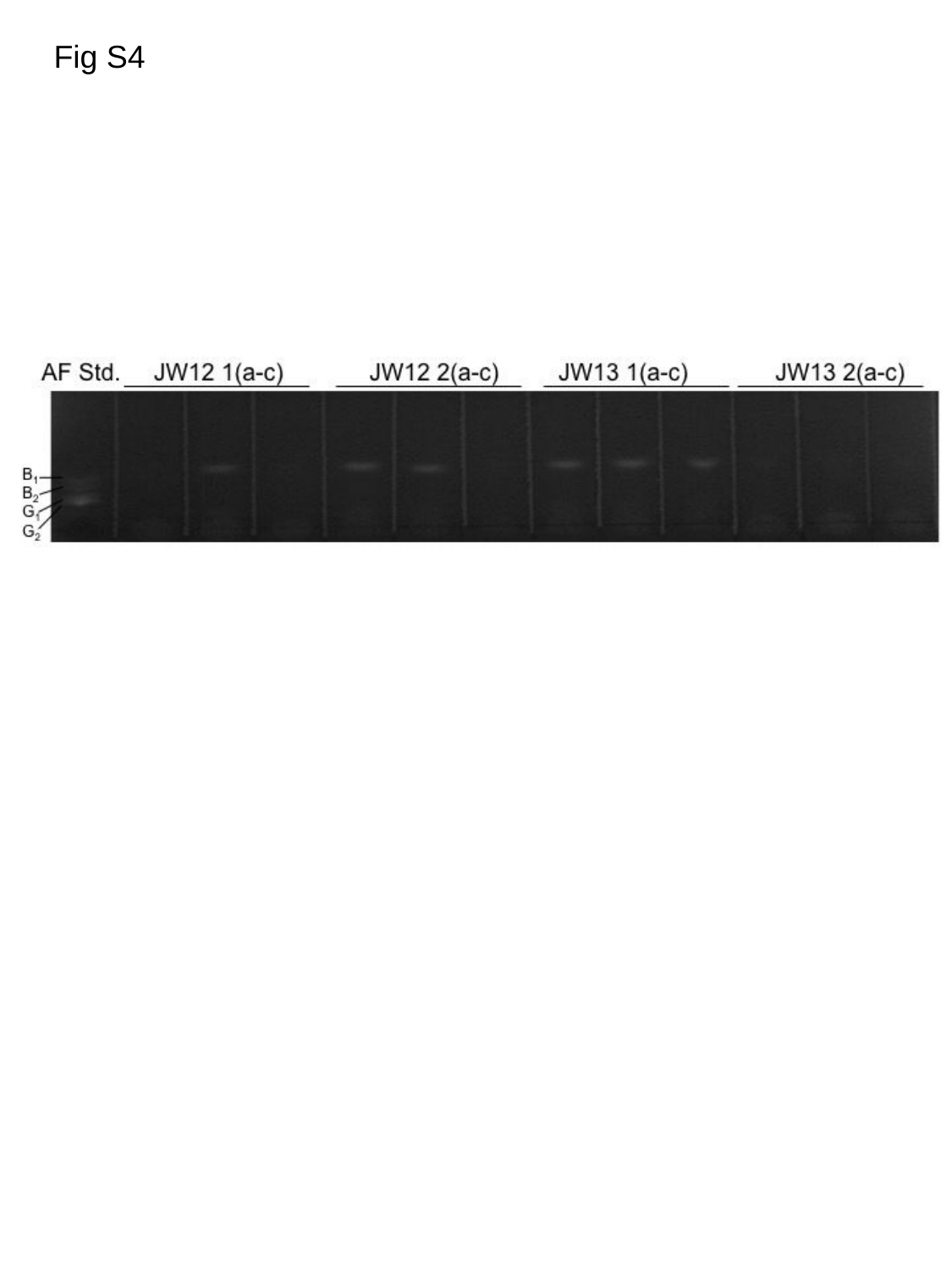

Fig S4

## Slide 5
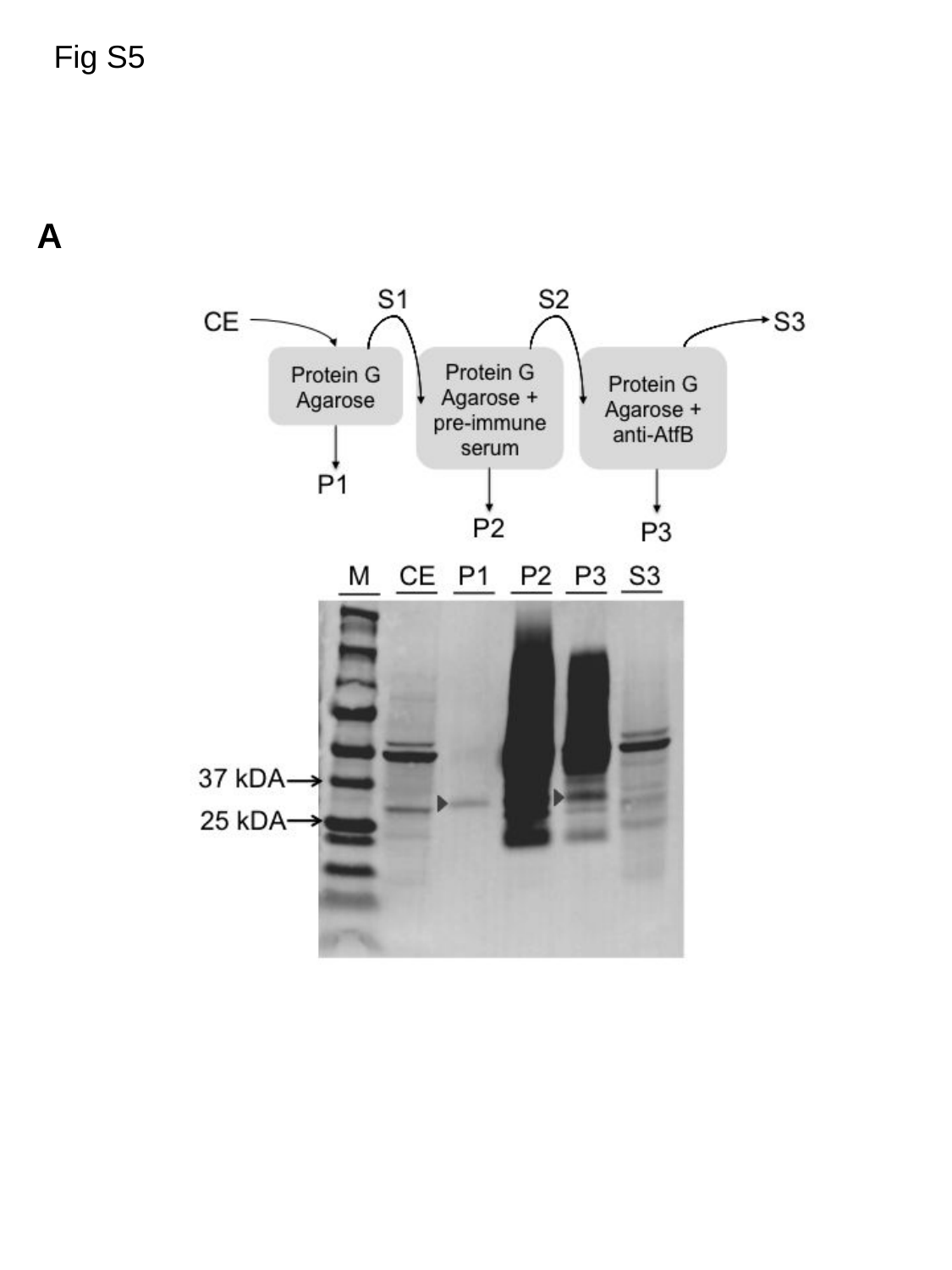

Fig S5
A

## Slide 6
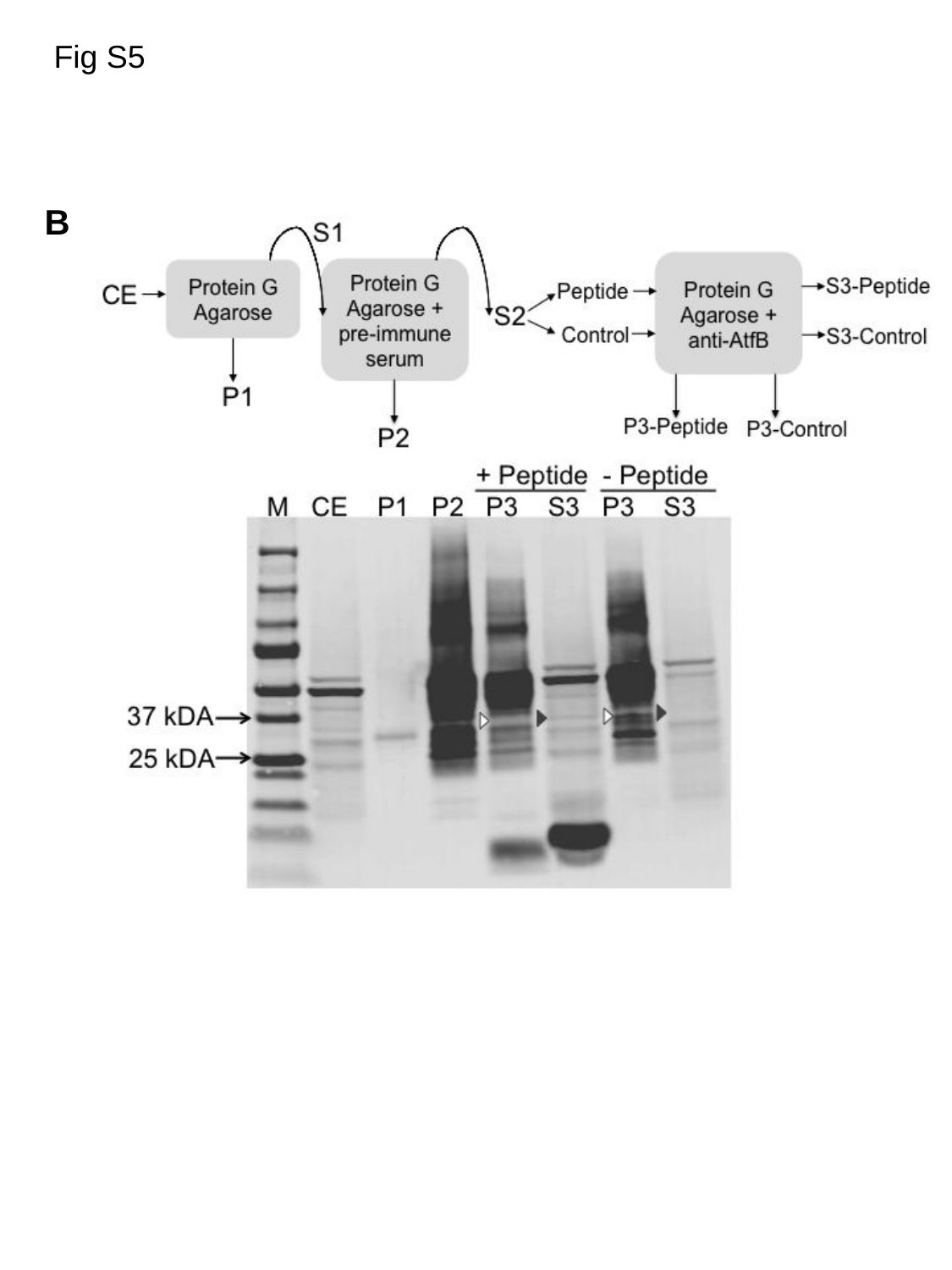

Fig S5
B

## Slide 7
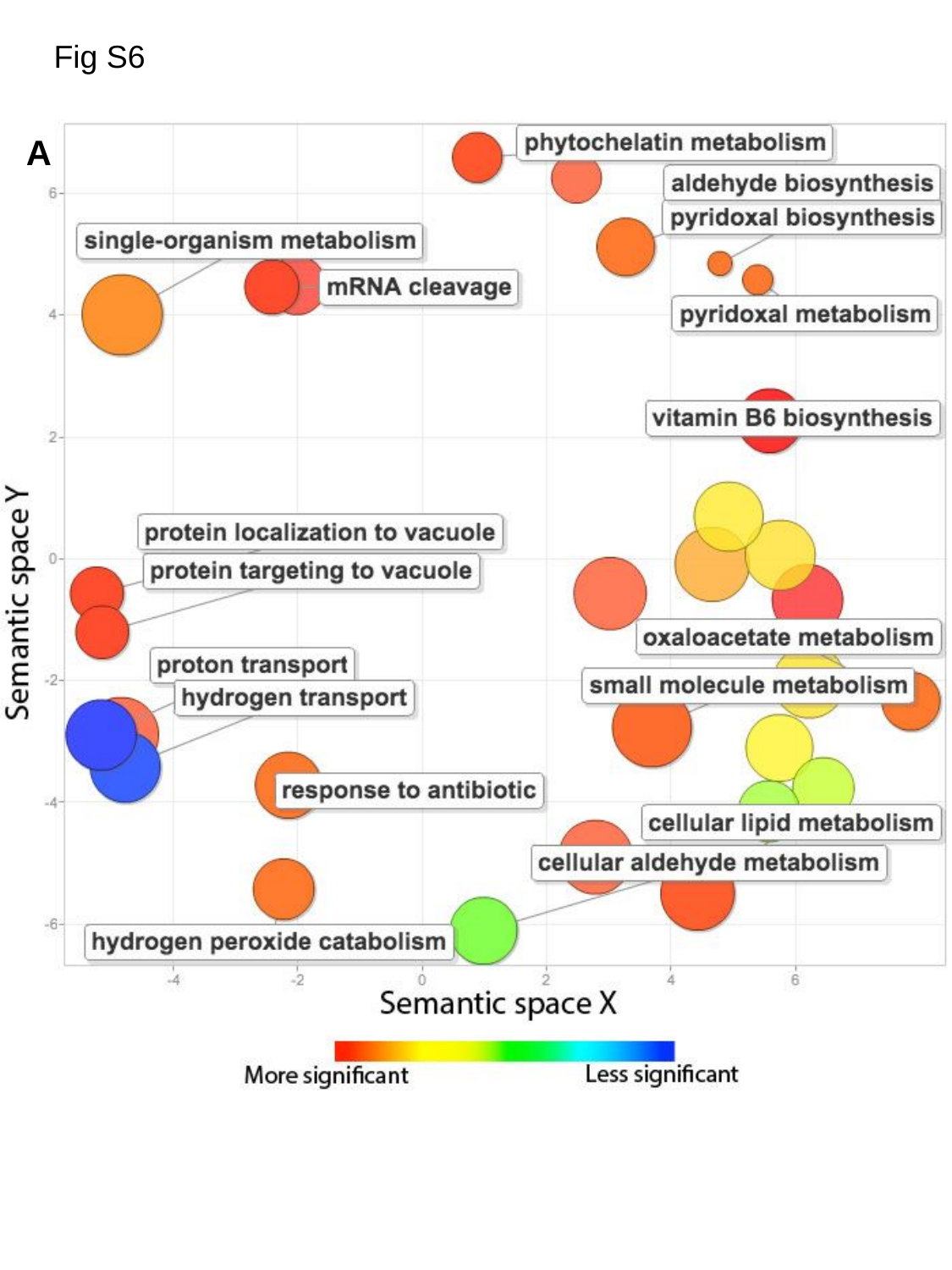

Fig S6
A

## Slide 8
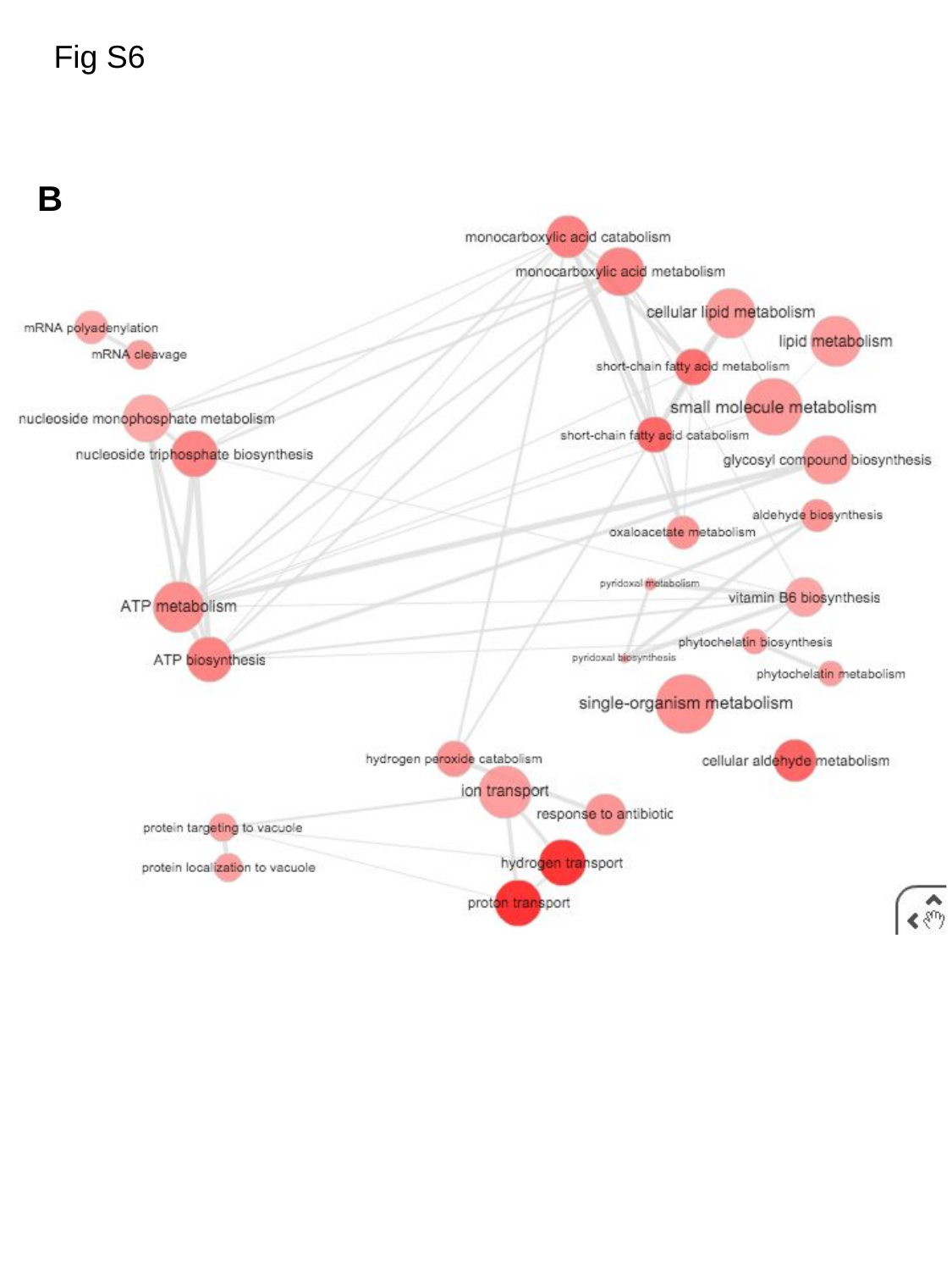

Fig S6
B

## Slide 9
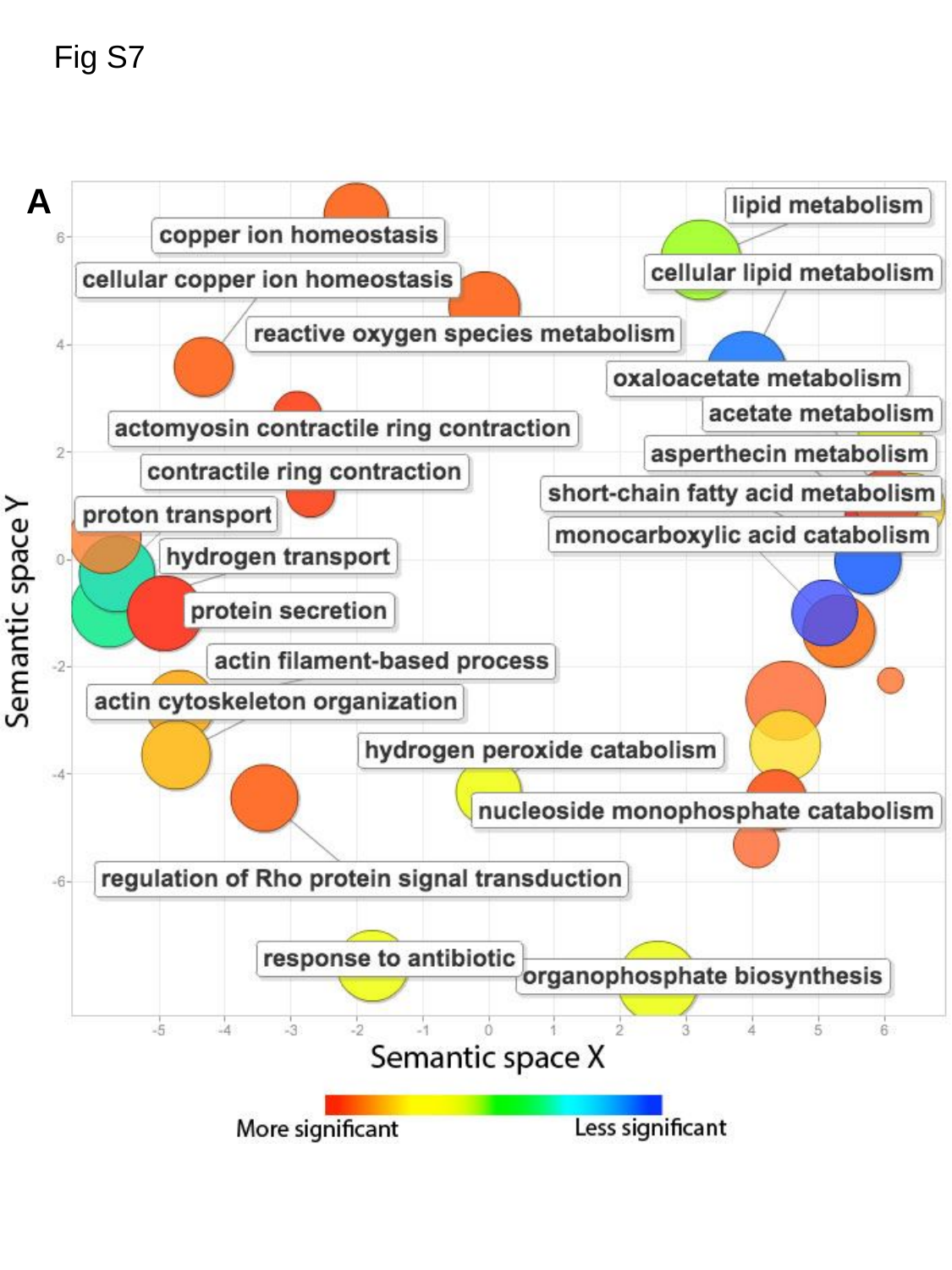

Fig S7
A

## Slide 10
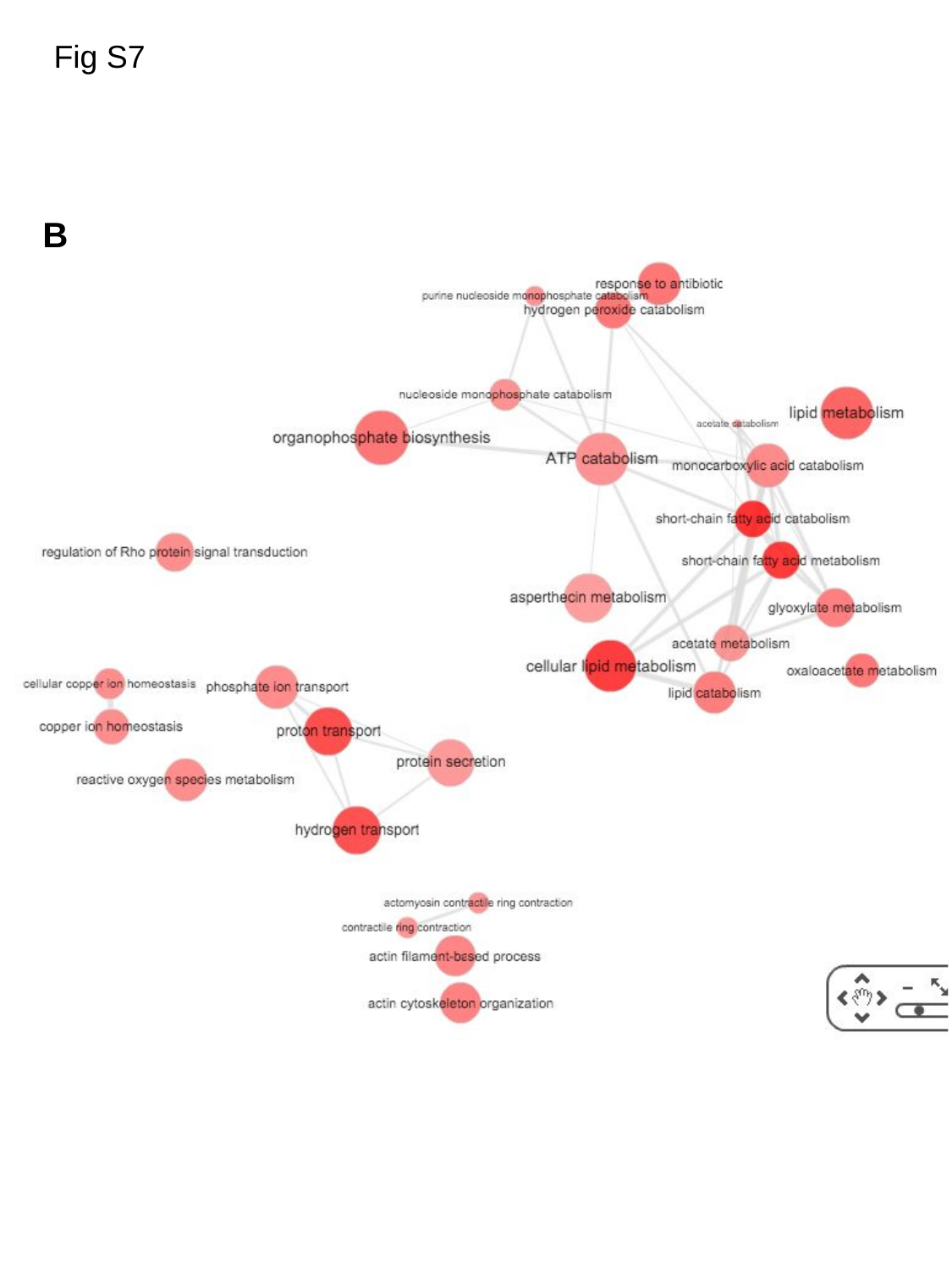

Fig S7
B

## Slide 11
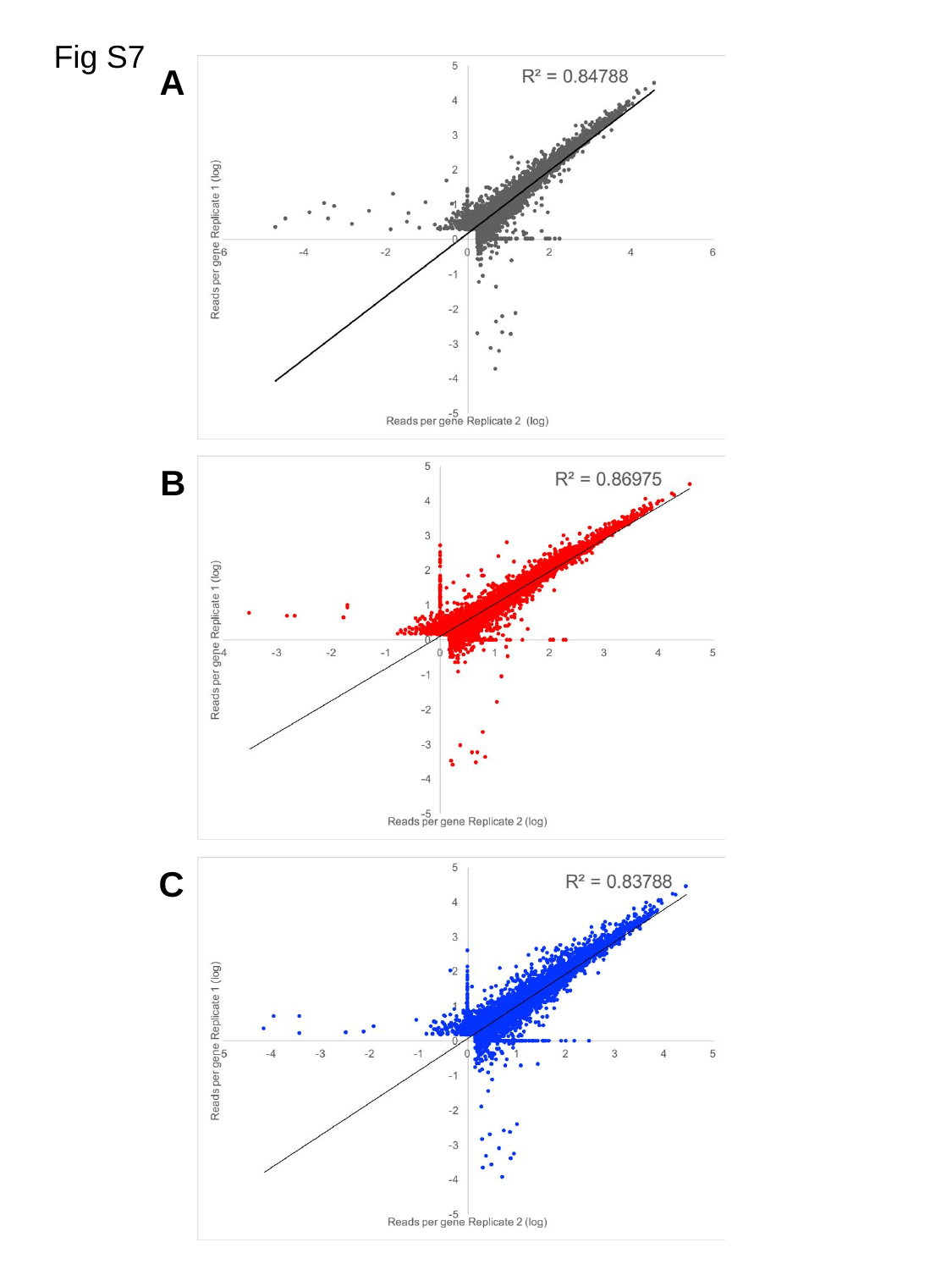

Fig S7
A
B
C
